# Viral Enrichment Methods Affect the Detection but Not Sequence Variation of West Nile Virus in Equine Brain Tissue

**DOI:** 10.3389/fvets.2018.00318

**Published:** 2018-12-18

**Authors:** Dhani Prakoso, Michael J. Dark, Anthony F. Barbet, Marco Salemi, Kelli L. Barr, Junjie J. Liu, Nanny Wenzlow, Thomas B. Waltzek, Maureen T. Long

**Affiliations:** ^1^Department of Comparative, Diagnostic and Population Medicine, College of Veterinary Medicine, University of Florida, Gainesville, FL, United States; ^2^Department of Infectious Diseases and Immunology, College of Veterinary Medicine, University of Florida, Gainesville, FL, United States; ^3^Department of Pathology, Immunology, and Laboratory Medicine, College of Medicine, University of Florida, Gainesville, FL, United States; ^4^Department of Biology, Baylor University, Waco, TX, United States; ^5^Department of Pathology and Microbiology, Faculty of Veterinary Medicine, University of Montreal, Saint-Hyacinthe, QC, Canada

**Keywords:** *West Nile virus*, arbovirus, equine, RNA extraction, viral enrichment, viral variation

## Abstract

West Nile virus (WNV), a small, positive sense, single stranded RNA virus continues to encroach into new locales with emergence of new viral variants. Neurological disease in the equine can be moderate to severe in the face of low to undetectable virus loads. Physical methods of virus enrichment may increase sensitivity of virus detection and enhance analysis of viral diversity, especially for deep sequencing studies. However, the use of these techniques is limited mainly to non-neural tissues. We investigated the hypothesis that elimination of equine brain RNA enhances viral detection without limiting viral variation. Eight different WNV viral RNA enrichment and host RNA separation methods were evaluated to determine if elimination of host RNA enhanced detection of WNV and increase the repertoire of virus variants for sequencing. Archived brain tissue from 21 different horses was inoculated with WNV, homogenized, before enrichment and separation. The protocols utilized combinations of low-speed centrifugation, syringe filtration, and nuclease treatment. Viral and host RNA were analyzed using real-time PCR targeting the WNV Envelope (E) protein and equine G3PDH to determine relative sensitivity for WNV and host depletion, respectively. To determine the effect of these methods on viral variation, deep sequencing of the E protein was performed. Our results demonstrate that additional separation and enrichment methods resulted in loss of virus in the face of host RNA depletion. DNA sequencing showed no significant difference in total sequence variation between the RNA enrichment protocols. For equine brain infected with WNV, direct RNA extraction followed by host RNA depletion was most suitable. This study highlights the importance of evaluating viral enrichment and separation methods according to tissue type before embarking on studies where quantification of virus and viral variants is essential to the outcome of the study.

## Introduction

During 1999, *West Nile virus* (WNV) was introduced to North America resulting in more than 49,794 human infections with 4% mortality, 28,063 equine infections, and over 100,000 avian population infections (1, 2). Since the North American encroachment, WNV has continued to emerge as multiple lineages with enhanced neurovirulence worldwide (3). In the US during 2012, a new subtype of the US lineage 1 virus emerged with a substantial increase in reporting of human and horse disease (4, 5). In Europe, a neurovirulent WNV lineage 2 has emerged, whereas historically this lineage caused a flu-like febrile illness in Africa (6). Diversification of WNV occurs through spontaneous mutation due to the poor proof-reading mechanisms inherent in positive-sense, single-stranded RNA viruses. This leads to the production of WNV variants are often associated with new tissues or cellular tropisms (7, 8) likely resulting in enhanced neurovirulence in humans and horses (9, 10). Viral diversity of WNV increases following infection of a new type of host cell (11), thus understanding viral variation within equine tissues can enhance our understanding of WNV pathogenicity in this host (12).

In model species such as the mouse and hamster, virus load is quite high and there are differences in the cellular composition and localization of virus in the central nervous system (CNS) compared to horses (13). Moreover, WNV lesions in human and equine brains are multifocal with virus concentrations ranging from as little as one hundred to one million plaque forming units (PFU)/mL (14–17). Horses develop titers of 10^1^ to 10^3^ PFU/mL in the blood and 10^4^ to 10^6.8^ in the brain (16). Often *in situ* methods of virus detection are unable to detect virus within affected tissues as part of post-mortem analysis. Low virus loads in plasma, extra-neural tissues, and the CNS limit our understanding of the pathogenesis of WNV in clinical symptomatic natural hosts.

Since WNV virus is difficult to detect in the equine, physical enrichment methods may be of use when extracting equine brain tissues. Yet, there are limited studies on physical enrichment techniques in a complex cellular milieu such as the equine brain (18, 19). The WNV genome is relatively small compared to other pathogens and the host, which increases the difficulty of performing consistent quantitative genomic data analysis using NGS technology within tissues with comparatively high host RNA (20). By increasing viral RNA concentration using a physical enrichment methods, sensitivity should be increased, which would reduce the cost of generating sequence data and save time for data analysis (19).

Several quick and established methods to separate viral RNA from host RNA include low-speed centrifugation, filtration, freeze-thaw, nuclease treatment, and combinations of these methods can also enhance extraction efficiency (19–22). The main principle of these methods is to separate the viral RNA from the host RNA without decreasing the overall amount of viral RNA or losing viral variants. Although many studies have been conducted to search for the best method to extract viral nucleic acid from different tissues of different organisms, none so far have focused on viral infection of the equine brain with WNV or any other arbovirus (18–22).

We investigated the hypothesis that elimination of equine brain RNA enhances viral detection without limiting viral variation. The goal of this study was to identify the effect of RNA enrichment methods on the amount and diversity of WNV RNA using inoculated fresh equine brain tissue. This study methodically investigated eight different methods to physically enrich viral RNA so that it would be concentrated for detection and sequencing without creating bias in virus variation.

## Materials and Methods

### Animals and Tissue Preparation

All work involving animals was performed in accordance with the University of Florida IACUC protocol: #201604559. Fresh brain tissue received for archiving from necropsies of horses donated for musculoskeletal and other non-infection extra-neural maladies were used. Tissues from 21 equines were immediately flash frozen in liquid nitrogen and stored at −80°C. In order to control for sample variation encountered in natural or experimental infection, brain tissues were experimentally inoculated. A computer based open source statistical software program (23) was used to calculate the samples size was necessary to detect differences between the RNA integrity in a natural population (Supplemental File 1). The same region of brain was inoculated and consisted of 150–200 mg of cerebrum just above the corona radiata. Each section was inoculated with 1.5 × 10^4^ PFU/mL of WNV NY99 (GenBank accession AF260967) using a blunt needle. This dose was chosen since horses develop titers of 10^4^−10^6.8^ PFU/mL in the brain via tissue culture, if detected at all in a specific region (16). This dose was based on gram weight of brain that would result in this range of virus. Tissues were then homogenized using beads under mechanical force (TissueLyser LT®; Qiagen, Germany) with an oscillation frequency of 50 Hz for 3.5 min in a cold rotor. Each homogenate was divided into eight aliquots for each type of virus enrichment and RNA extraction method.

### RNA Extraction, Separation, and Enrichment

**Figure 1:**
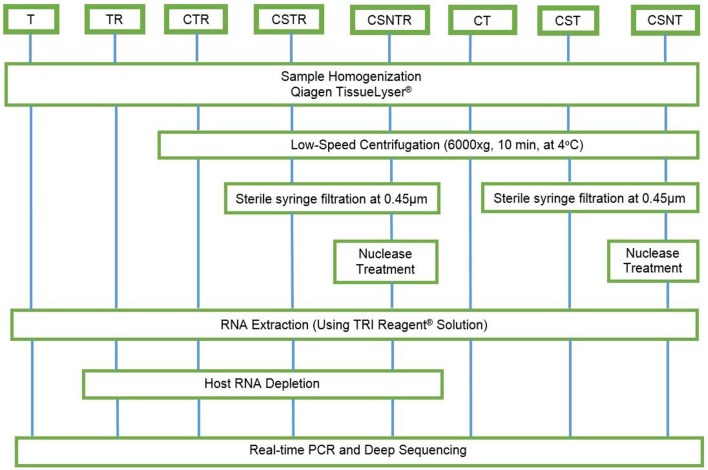
Workflow for the preparation of equine brain tissue for testing different viral enrichment methods. The eight treatments consisted of T, TRI Reagent® RNA extraction only; TR, TRI Reagent® RNA extraction followed by host RNA depletion; CTR, low speed centrifugation followed by TRI Reagent® RNA extraction and host RNA depletion; CSTR, low speed centrifugation followed by sterile syringe filtration, TRI Reagent® RNA extraction, and host RNA depletion; CSNTR, low speed centrifugation followed by sterile syringe filtration, nuclease treatment, TRI Reagent® RNA extraction, and host RNA depletion; CT, low speed centrifugation followed by TRI Reagent® RNA extraction; CST, low speed centrifugation followed by sterile syringe filtration and TRI Reagent® RNA extraction; CSNT, low speed centrifugation followed by sterile syringe filtration, nuclease treatment, and TRI Reagent® RNA extraction.

Eight combinations of virus enrichment and RNA separation methods were used. These included guanidium/phenol/chloroform extraction (T), host RNA depletion (R) using a commercial kit (RiboMinus™ Eukaryote System v2, Life Technologies, Carlsbad, CA), low speed centrifugation (C) at 6000 × g at 4°C for 10 m (19), sterile syringe filtration (S) at 0.45 μm (Thermo Scientific, Waltham, MA), and nuclease (N) treatment (0.1 U/μL Turbo DNAse, Life Technologies, Carlsbad, CA). Combinations of these resulted in eight separate groups consisting of T, TR, CTR, CSTR, CSNTR, CT, CST, and CSNT groups (Figure 1). All groups used standard phase separation RNA extraction (TRI Reagent®, Life Technologies, Carlsbad, CA). The WNV viral RNA was evaluated using microfluidics spectrophotometry using the RNA integrity number (RIN) (Agilent Bionalyzer®; Agilent, Santa Clara, CA) for quality assessment (24).

### Real-Time PCR

RNA was converted into cDNA using a commercial kit (High Capacity RNA to cDNA; Applied Biosystems, Foster City, CA) and addition of oligo dT's (Thermo Scientific, United Kingdom) according to the manufacturers' protocols. The copy numbers of WNV Envelope (E) gene and equine G3PDH gene were measured using real-time PCR (Table 1) as previously described (25–27). Real-time PCR analysis was performed using a commercial real-time PCR machine and associated chemistries (ABI Fast 7500 system machine; Applied Biosystems, Foster City, CAs). Each reaction consisted of 10 μL master mix (TaqMan Fast Advanced® Master Mix; Foster City, CA) and 2 μL of cDNA sample in a final volume of 20 μL (25, 28). The cycling conditions were 50°C for 2 min, 95°C for 20 s, and then 40 cycles of 95°C for 3 s and 60°C for 30 s during which the fluorescence data were collected. Each assay included standard curves based on the WNV E gene plasmid and G3PDH synthetic DNA (Biosearch Technologies, Petaluma, CA) with known copy number. Positive and negative controls were also included in each assay.

**Table 1:** Primers and probes sets used to detect WNV and G3PDH.

**Target**	**Real-time PCR primers and probes 5**^****′****^**-3**^****′****^	
WNV Envelope^a^	Forward	TCAGCGATCTCTCCACCAAAG
	Reverse	GGGTCAGCACGTTTGTCATTG
	Probe	FAM-TGCCCGACCATGGGAGAAGCTC–BHQ-1
Equine G3PDH^b^	Forward	TAAACGGATTTGGCCGTATTGG
	Reverse	TGAAGGGGTCATTGATGGCG
	Probe	FAM-CAGGGCTGCTTTTAACTCTGGCAAAGTGGA-BHQ-1
		**Sequencing primers 5′-3′**
WNV Envelope	Forward	GCCAAATTTGCCTGCTCTAC
	Reverse	ACCAAGAACGTCTTTGTTCCA

a *Publication reference: Kauffman et al. (25) and Lanciotti et al. (26)*.

b *Publication reference: Peters et al. (27)*.

### DNA Sequencing

Four samples were randomly selected from each treatment group. Because WNV is present in low virus quantities in natural infection, PCR amplification prior to sequencing was performed to simulate conditions that may be need for phylogenetic studies of clinical WNV horses. Fragments of cDNA were amplified using conventional PCR (Table 1). The PCR reaction consisted of 12.5 μL of a commercial master mix (Q5® High-Fidelity 2X Master Mix; New England BioLabs, Ipswich, MA), 0.5 μM of each primer, 3 μL of template cDNA sample in 25 μL final volume. The cycling conditions consisted of 98°C for 30 s, 35 cycles of 98°C for 10 s, 65°C for 30 s, 72°C for 30 s, followed by final extension step 72°C for 2 min. The PCR amplicons were isolated in a 2% agarose gel via electrophoresis and purified using a commercial kit (Wizard® SV Gel and PCR Clean-Up System; Promega, Madison, WI). A commercial sequencing platform was used (MiSeq instrument; Illumina, San Diego, CA) and libraries were prepared using the manufacturer's protocols and chemistries (Nextera XT DNA Sample Preparation Kit; Illumina, San Diego, CA). The quality and quantity of each DNA library was verified as previously described.

### Sequence and Variant Analysis

Sequence data files, consisting of forward and reverse sequences were mapped and filtered for quality, size, and parity (Galaxy Project, Illumina) (29). Briefly, all sequences were converted to FASTQ files. The files were analyzed using a FASTQ manipulation computer program (Groomer tool and the FASTQ Interlacer tool; Galaxy) to join paired-end FASTQ reads of the forward mates with the reverse mates into a single sequence file. Forward mates were alternated with their reverse mates, all sequences without mates were filtered out (30). The 3′-end or the 5′-end of reads were trimmed (Sickle tool, Galaxy) using quality and length thresholds of 30 and 125 bp, respectively (31). Sequences were aligned with the reference sequence WNV strain NY99 (GenBank accession AF260967; Bowtie2, Galaxy) (32). Sequences were sorted in coordinate order (Sortsam, Galaxy) and a variant inference software program (V-Phaser 2.0) for viral populations was used to calculate the strand-bias *p*-value, type of variants, and frequency of variants (33).

### Statistical Analysis

WNV E protein and G3PDH gene expression, RIN, mapped reads, and total variation, were compared between all treatments groups using Welch's ANOVA where *p* < 0.05 was considered significant (Minitab® 17 software, Minitab Inc., State College, PA). The Games-Howell pairwise comparison test was used to detect differences between groups. In order to analyze whether the frequency of variation was affected by an extraction method, variation was grouped into one of four classifications based on the frequency of observation for that variant, with levels defined as 0–0.5, 0.5–1, 1–5, and 5–50% of the total variants. Differences were then analyzed by multiple regression analysis (*p* < 0.05) with treatment and frequency classification as predictor or dependent variables.

## Results

### Viral Enrichment Results in Decreased RNA Integrity

**Figure 2:**
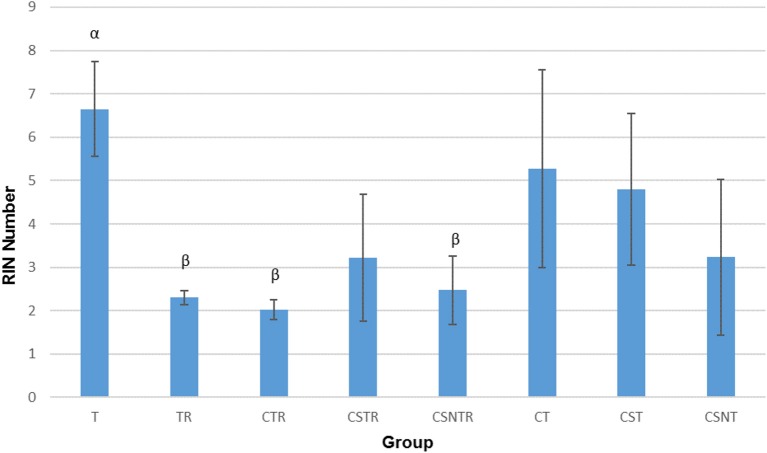
The effect of eight RNA enrichment methods on RNA integrity. Microfluidic based spectrophotometry (Agilent Bioanalyzer) was performed on the RNA derived from each horse (21) in each treatment group and results are graphed as the mean RNA integrity measurement (RIN). Significance *p* < 0.05. T, TRI Reagent® RNA extraction only; TR, TRI Reagent® RNA extraction followed by host RNA depletion; CTR, low speed centrifugation followed by TRI Reagent® RNA extraction and host RNA depletion; CSTR, low speed centrifugation followed by sterile syringe filtration, TRI Reagent® RNA extraction, and host RNA depletion; CSNTR, low speed centrifugation followed by sterile syringe filtration, nuclease treatment, TRI Reagent® RNA extraction, and host RNA depletion; CT, low speed centrifugation followed by TRI Reagent® RNA extraction; CST, low speed centrifugation followed by sterile syringe filtration and TRI Reagent® RNA extraction; CSNT, low speed centrifugation followed by sterile syringe filtration, nuclease treatment, and TRI Reagent® RNA extraction.

The RIN is an algorithm for assessing the integrity values of the RNA (24). The RIN displayed a significant decrease in concert with increased viral enrichment steps (*p* = 0.002). The highest mean RIN was detected in group T (RIN 6.65) and the lowest mean RIN was detected in group CTR (RIN 2.025; Figure 2). There was a significant difference between the treatment groups and the RIN (*p* = 0.002). Low average RIN numbers were measured from treatment groups TR, CTR, and CSNTR with average RINs of 2.3, 2.024, and 2.475, respectively. High average RIN numbers were measured from groups T (RIN 6.65) and CT (RIN 5.275), while the CSNT, CSTR, and CST treatment groups showed intermediate RNA quality with RINs ranging from 3.2 to 4.8.

The treatment groups with RNA depletion with selective depletion of host ribosomal RNA (group TR, CTR, CSTR, and CSNTR) had significantly lower RIN numbers compared to the groups which did not have the host RNA depletion treatment (group T, CT, CST, and CSNT) (*p* = 0.001).

### Viral Enrichment Methods Decrease Viral RNA Detection

**Figure 3:**
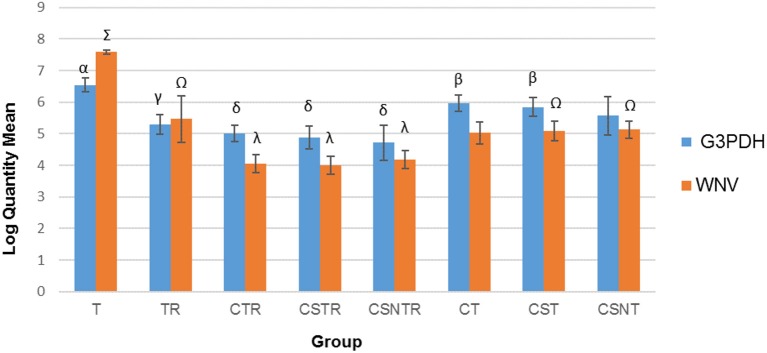
The effect of eight RNA enrichment techniques on virus quantity compared to host depletion. Real-time rtPCR was performed on the RNA derived from each horse (21) in each treatment group and the results are graphed as the mean copy number (log_10_) of the WNV Envelope gene and the equine G3PDH. Significance *p* < 0.05. T, TRI Reagent® RNA extraction only; TR, TRI Reagent® RNA extraction followed by host RNA depletion; CTR, low speed centrifugation followed by TRI Reagent® RNA extraction and host RNA depletion; CSTR, low speed centrifugation followed by sterile syringe filtration, TRI Reagent® RNA extraction, and host RNA depletion; CSNTR, low speed centrifugation followed by sterile syringe filtration, nuclease treatment, TRI Reagent® RNA extraction, and host RNA depletion; CT, low speed centrifugation followed by TRI Reagent® RNA extraction; CST, low speed centrifugation followed by sterile syringe filtration and TRI Reagent® RNA extraction; CSNT, low speed centrifugation followed by sterile syringe filtration, nuclease treatment, and TRI Reagent® RNA extraction.

The number of transcripts detected by real-time PCR decreased with increased processing while a concomitant decrease in host RNA was achieved (Figure 3). The highest copy number for both WNV E protein and G3PDH was detected in the T treatment group which was significantly elevated compared to all other treatment groups (*p* = 0.001). The treatment groups with low copy numbers for both WNV E protein and G3PDH were from groups CTR, CSTR, and CSNTR (Figure 3). Groups T and TR had lower G3PDH copy numbers than the WNV E protein copy numbers of the same group, while all other groups displayed the opposite (Figure 3).

### Viral Enrichment Methods Do Not Affect Sequence Data

**Figure 4:**
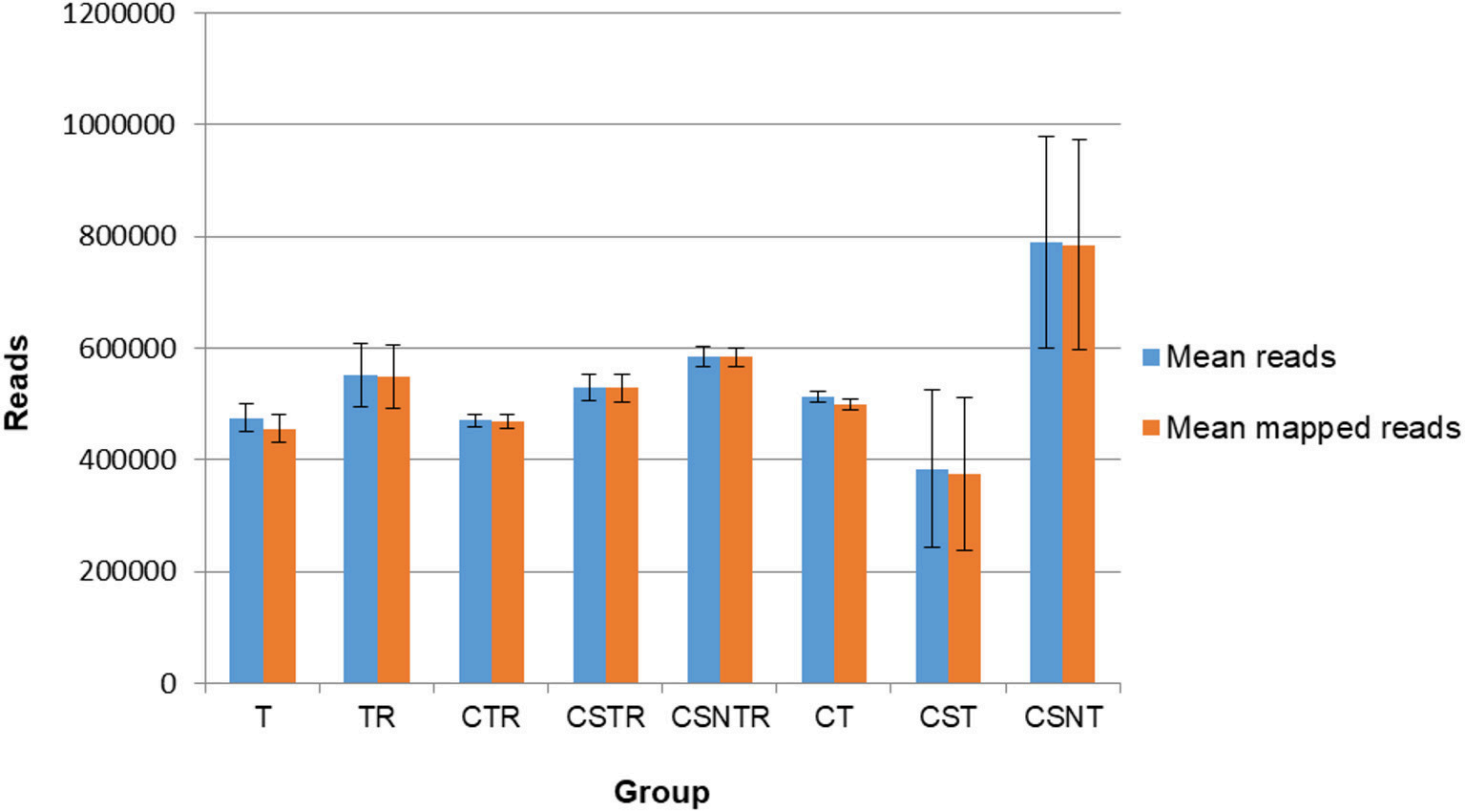
The impact of RNA enrichment on the number of reads obtained with deep sequencing. Deep sequencing (Illumina) was performed on the RNA derived from each horse in each treatment group and results are graphed as the average number of reads (±standard deviation) and mapped reads (±standard deviation). Significance *p* < 0.05. T, TRI Reagent® RNA extraction only; TR, TRI Reagent® RNA extraction followed by host RNA depletion; CTR, low speed centrifugation followed by TRI Reagent® RNA extraction and host RNA depletion; CSTR, low speed centrifugation followed by sterile syringe filtration, TRI Reagent® RNA extraction, and host RNA depletion; CSNTR, low speed centrifugation followed by sterile syringe filtration, nuclease treatment, TRI Reagent® RNA extraction, and host RNA depletion; CT, low speed centrifugation followed by TRI Reagent® RNA extraction; CST, low speed centrifugation followed by sterile syringe filtration and TRI Reagent® RNA extraction; CSNT, low speed centrifugation followed by sterile syringe filtration, nuclease treatment, and TRI Reagent® RNA extraction.

Sequence data were analyzed for mean reads and mean number of reads that mapped to the reference sequence (Figure 4). Although the CSNT group had the highest number of mean reads, there was no significant difference between treatment groups and mapped reads (*p* = 0.103) (Figure 4). The CST group had the fewest reads and mapped reads compared to the other treatment groups. For both of the CST and CSNT treatments, variation increased dramatically in these treatment groups. The amount of reads in the CST group were between 18.42 and 51.45% lower than other groups (Figure 4). The agreement of obtained sequences with the reference sequence was almost equal to the mean number of reads, which reflects a high degree of fidelity and consistency in cartridge loading.

**Figure 5:**
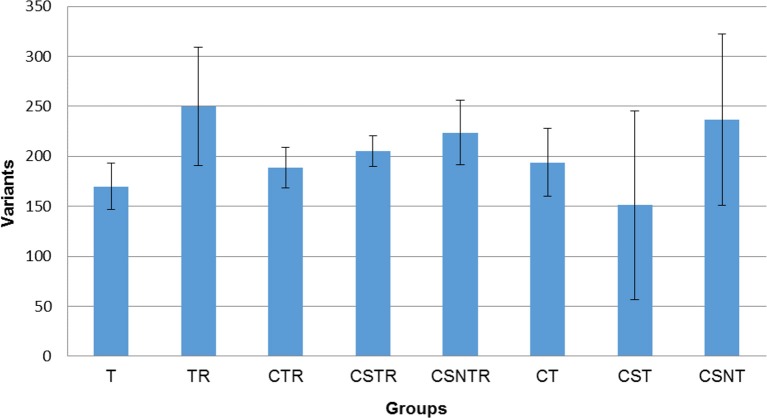
The effect of RNA enrichment on WNV sequence variation. Deep sequencing (Illumina) was performed on the RNA derived from each horse and results are graphed as the mean (± standard deviation) of total number of variants from each horse in each treatment group. Results were compared using one-way ANOVA (*p* < 0.05). The variants were calculated by counting the amount of SNV (Single Nucleotide Variant) and LPV (Length Polymorphism Variant) from the V-Phaser 2.0 software output in each sample. T, TRI Reagent® RNA extraction only; TR, TRI Reagent® RNA extraction followed by host RNA depletion; CTR, low speed centrifugation followed by TRI Reagent® RNA extraction and host RNA depletion; CSTR, low speed centrifugation followed by sterile syringe filtration, TRI Reagent® RNA extraction, and host RNA depletion; CSNTR, low speed centrifugation followed by sterile syringe filtration, nuclease treatment, TRI Reagent® RNA extraction, and host RNA depletion; CT, low speed centrifugation followed by TRI Reagent® RNA extraction; CST, low speed centrifugation followed by sterile syringe filtration and TRI Reagent® RNA extraction; CSNT, low speed centrifugation followed by sterile syringe filtration, nuclease treatment, and TRI Reagent® RNA extraction.

In order to determine if viral enrichment treatments could enhance the ability to detect the number of virus variations in a sample, the mean total virus variation of each treatment group was analyzed (V-Phaser 2 software). The TR, CSNT, and CSNTR groups had the highest mean total virus variation but this difference was not significant (*p* = 0.185) (Figure 5). The CST group had the fewest variants with an average of 159 variants, which was 39% lower than the highest group, TR that had an average of 249.75 variants.

**Figure 6:**
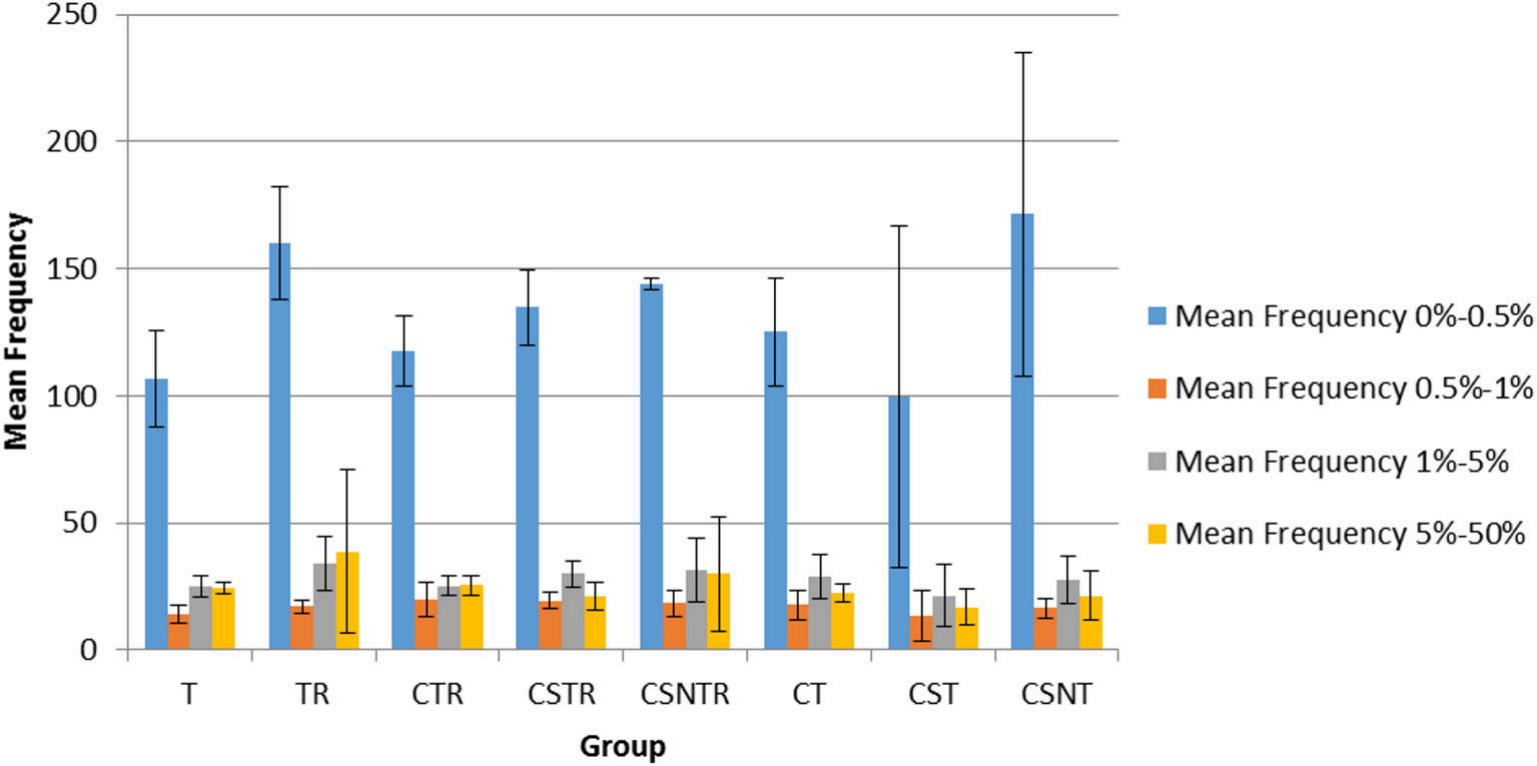
The impact of RNA enrichment and host RNA depletion on the frequency of WNV sequence variants. Deep sequencing (Illumina) was performed on the RNA derived from each horse and each variant was classified into one of four classifications based on the frequency of observation for that variant, with levels defined as 0–0.5, 0.5–1, 1–5, and 5–50% of the total variants (V-Phaser 2.0). The bar graphs represent the mean and standard deviation and the data was analyzed by multiple regression (*p* < 0.05). T, TRI Reagent® RNA extraction only; TR, TRI Reagent® RNA extraction followed by host RNA depletion; CTR, low speed centrifugation followed by TRI Reagent® RNA extraction and host RNA depletion; CSTR, low speed centrifugation followed by sterile syringe filtration, TRI Reagent® RNA extraction, and host RNA depletion; CSNTR, low speed centrifugation followed by sterile syringe filtration, nuclease treatment, TRI Reagent® RNA extraction, and host RNA depletion; CT, low speed centrifugation followed by TRI Reagent® RNA extraction; CST, low speed centrifugation followed by sterile syringe filtration and TRI Reagent® RNA extraction; CSNT, low speed centrifugation followed by sterile syringe filtration, nuclease treatment, and TRI Reagent® RNA extraction.

The frequency of variation was categorized into four levels including 0–0.5, 0.5–1, 1–5, and 5–50%. In all groups, the mean number of observations was statistically higher in the 0–0.5% frequency classification across all treatment groups (Figure 6) and there was no effect of extraction treatment on the mean frequency of between each frequency classification.

## Discussion

The goal of this study was to identify RNA enrichment methods for the WNV infected equine brain with the hypothesis that the viral enrichment would increase the detection sensitivity and that viral diversity would be maintained or enhanced. The overall goal of this research was to determine which technique would be most applicable to extraction of tissues from the clinically diseased horse. We purposely chose this experimental design to control for the variation expected if experimentally or naturally infected animals were used, in order to establish a technical protocol for intrahost phylogenetic studies. This allowed us to determine, without bias, the best methods for obtaining consistent virus RNA for downstream sequencing experiments to contribute to the newly emerging field of phyloanatomy (34, 35). In both natural and experimental infection, several publications have demonstrated that WNV infection in the equine brain and spinal cord is multifocal (14–17). In addition, lesions and viral load is quite variable across regions. Thus, the goal was establish optimal detection methods for virus specifically in the equine brain.

Real-time PCR results showed that phase extraction only resulted in the highest WNV E protein gene and equine G3PDH copy numbers. Clearly, extraction technique could affect the sensitivity of detection of WNV nucleic acids, which has implications for diagnostic testing. What is unknown is if viral sensitivity is altered with incorporation of various commercial RNA extraction kits.

The amount of WNV E protein and equine G3PDH decreased with fewer enrichment treatments likely indicating a lack of separation of virus from host tissues. Since this model used inoculation without infection, a portion of intracellular virus would also likely be depleted with more separation with more separation with actual infection. Additive methods such as low speed centrifugation, syringe filtration, nuclease treatment, and host RNA depletion had no obvious improvement in terms of viral variant analysis or for detection purposes. Even host cell depletion alone resulted in a profound loss of viral RNA. However, these results must be interpreted in the context of the specific virus and sample.

Groups TR, CTR, CSTR, and CSNTR had lower WNV E protein and G3PDH copy numbers per microliter compared to the other four treatment groups. The difference between these treatment groups was the host RNA depletion treatment. The host RNA depletion kit used was designed for human, mouse, and rat samples and may not work as well in equine or neural tissues. For virus detection, group T was the best method as the DNA copy number for WNV E protein and G3PDH amount was the greatest compared with the other treatment groups.

WNV virus sequence variations were significantly different between treatment groups and the TR group had the highest average number of variants detected in the samples. Group T could be considered for RNA extraction since it had the highest WNV E protein RNA recovery and will be an important consideration where virus is minimally detectable. Notable is the amount of variation in sequence introduced in the CSTR and CSNTR, which will have an effect on the ability to detect significant differences in variation between samples.

Methods that separate host materials from pathogens have been investigated for RNA viruses and are important for viral discovery and deep sequencing studies. However, there are few studies that provide conclusive evidence that host genome depletion enriches for viral RNA in a manner that sensitivity of viral detection is enhanced or that investigation of viral variation is improved for brain (18–22). Some available methods using different viruses such as adenovirus, influenza A virus, enterovirus, lyssavirus, hantavirus, hepatitis B virus, hepatitis C virus, canine parvovirus, and canine adenovirus show that viral enrichment methods are specific to viruses and tissue types (18–22).

We incorporated PCR into this protocol because prior literature indicates inconsistent results in sequencing output in the face of positive real time rtPCR results in equine WNV infection (35). This is especially important for WNV disease in horses where virus is highly variable regionally in the CNS. Previous studies utilized nested PCR before sequencing (36) and nested PCR techniques are still being utilized for rapid genotyping of WNV virus in equine tissues and plasma (37). Deep sequencing may obviate this requirement but given the host RNA to viral RNA ratio, host viral sequences would have become the predominant output which could limit the ability to detect rare variants. Nonetheless, PCR could create bias due to introduced errors, hence high fidelity reagents must be used. In this particular data, a high degree of low frequency variants were detected which are essential for examining viral variation. This structure was maintained across treatments and PCR reactions. Deep sequencing without prior amplification may be efficient for viruses that reside in high quantity in plasma or more uniformly in tissues.

Studies comparing direct deep sequencing with prior amplification would be required to investigate the effect of PCR on viral variation for this particular virus in equine brain. For this type of study, an experimental design incorporating clinical and/or experimentally infected horses would be appropriate.

## Conclusion

This study highlights the risk of using enrichment treatments without consideration of the virus target and the sample type. Additive methods beyond host cell depletion had no obvious improvement in terms of viral variant analysis or viral detection; for example, host cell RNA depletion resulted in a profound loss of viral RNA. Evaluation of viral enrichment and separation method appropriate for the sample type is important for viral detection and later sequencing.

## Ethics Statement

This study was carried out in accordance with the recommendations of the University of Florida Institutional Animal Care and Use Committee.

## Author Contributions

DP, TW, MS and ML designed the study. DP, JL, and NW performed necropsy of the equines and confirmation of post-mortem findings. DP performed RNA extraction, real-time PCR testing, and sequencing. DP, ML, and KB performed data auditing and analysis. TW provided sequencing service and consultation. MD and AB assisted in sequencing data analysis. DP wrote the manuscript draft while the other authors edited the manuscript.

### Conflict of Interest Statement

The authors declare that the research was conducted in the absence of any commercial or financial relationships that could be construed as a potential conflict of interest.
