# Supplementary material for: Viral Enrichment Methods Affect the Detection but Not Sequence Variation of West Nile Virus in Equine Brain Tissue
Source: Front Vet Sci. 2018 Dec 18;5:318. doi: 10.3389/fvets.2018.00318 (PMC6305279; doi:10.3389/fvets.2018.00318)
Supplement: Supplementary file 1 [file Data_Sheet_1.pdf]

Supplemental File 1

Viral Enrichment Methods Affect the Detection but not Sequence Variation of West Nile virus in Equine Brain Tissue

Dhani Prakoso[1], Michael J. Dark[1], Anthony F. Barbet[2], Marco Salemi[3], Kelli L. Barr[4], Junjie J. Liu[1], Nanny Wenzlow[5], Thomas B. Waltzek[2], Maureen T. Long[1*]

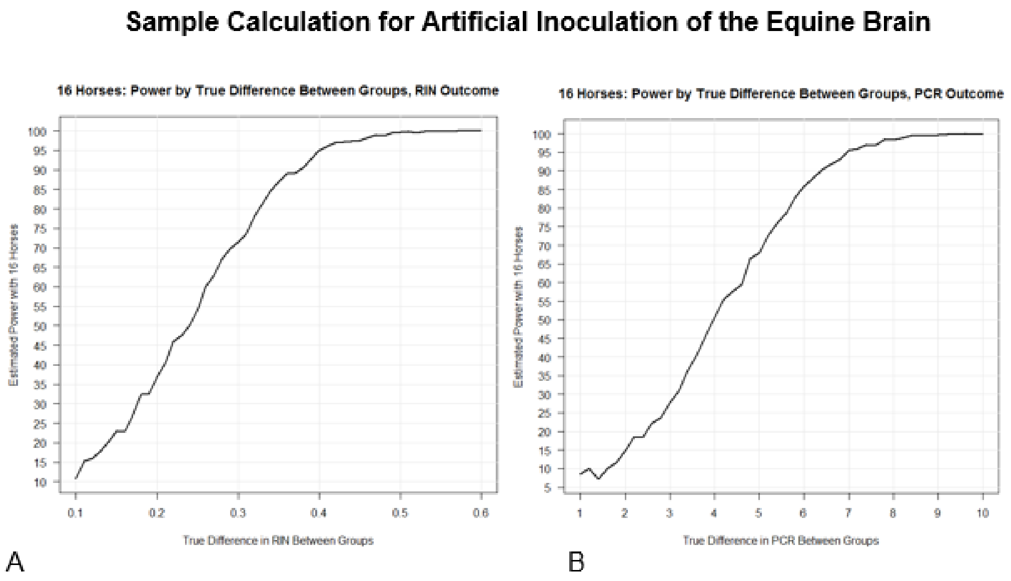

Figure A-1. Sample calculation for artificial inoculation of the equine brain to simulate effect of treatment on viral detection and diversity. Power analysis for calculating the true difference between groups of treatments for sixteen horses, four samples per horse, with calculation of host depletion and detection of virus (outcomes: RIN and real-time PCR detection of G3PDH and WNV). The curves display the power we expected with sixteen horses with a significance of $p < 0.05$. According to the graphs above, for RIN outcome (A), if the greatest difference between any two of the groups was equal to 0.34 units, then we have approximately an 80% chance of getting $p < 0.05$. For detection of differences in G3PDH and WNV (B), in order to have 80% power, any two of the groups will need to vary by at least 5.8 units.
